# Association Between Smoking Hookahs (Shishas) and Higher Risk of Obesity: A Systematic Review of Population-Based Studies

**DOI:** 10.3390/jcdd6020023

**Published:** 2019-06-16

**Authors:** Reem Baalbaki, Leila Itani, Lara El Kebbi, Rawan Dehni, Nermine Abbas, Razan Farsakouri, Dana Awad, Hana Tannir, Dima Kreidieh, Dana El Masri, Marwan El Ghoch

**Affiliations:** Department of Nutrition and Dietetics, Faculty of Health Sciences, Beirut Arab University, P.O. Box 11-5020 Riad El Solh, Beirut 11072809, Lebanon; reembaalbaki1998@gmail.com (R.B.); l.itani@bau.edu.lb (L.I.); larakebbe@gmail.com (L.E.K.); rawan-dehni@hotmail.com (R.D.); nermine.abbas7@gmail.com (N.A.); razane.fa@hotmail.com (R.F.); dana_awad@outlook.com (D.A.); hana.tannir@bau.edu.lb (H.T.); d.kraydeyeh@bau.edu.lb (D.K.); dana.masri@bau.edu.lb (D.E.M.)

**Keywords:** obesity, hookah, overweight, shisha, abdominal obesity, weight management, treatment

## Abstract

The American Heart Association has published a scientific statement on the effect of hookah smoking on health outcomes; nevertheless, hookah smoking continues to be popular worldwide, especially among the young. Recent reports mention a potential link between hookah smoking and obesity; however, uncertainties still surround this issue. The aim of the current study was to conduct a systematic review to clarify whether hookah smoking is associated with a higher risk of obesity among the general population. This study was conducted in compliance with the preferred reporting items for systematic reviews and meta-analyses (PRISMA) guidelines, and data were collated by means of a meta-analysis and a narrative synthesis. Of the 818 articles retrieved, five large-population and low-bias studies comprising a total of 16,779 participants met the inclusion criteria and were reviewed. All included studies reported that, regardless of gender, hookah smoking increases the risk of obesity among all ages and observed an association between the two after a correction for several confounders or reported a higher prevalence of obesity among hookah smokers. This was confirmed by the meta-analysis. Therefore, hookah smoking seems to be associated with a higher risk of obesity. Public health policymakers should be aware of this for the better management of obesity and weight-related comorbidities.

## 1. Introduction

The hookah, also known as a water pipe, narghile, arghile, or shisha, was invented in the 16th century as an attempt to purify smoke through water [1]. Nowadays hookah smoking is becoming popular in developing countries as well as in Western countries, especially among the young [2,3,4]. In fact, many hookah smokers consider this practice less harmful than smoking cigarettes because of the misconception that inhaling smoke containing fruit flavours, (i.e., apple, orange, grapes, etc.) through hookah water is less toxic [5]. Strong evidence supports the association between hookah smoking and several chronic diseases as well as a high risk of cancer [6,7,8,9,10,11,12,13,14] to the extent where it is considered a serious public health problem. This caused the American Heart Association to issue a scientific statement on hookah smoking and the increased risk of cardiovascular disease [15].

On the other hand, obesity is another increasing health problem. It is becoming one of the most serious conditions worldwide, known to be associated with several comorbidities that lead to an increase in disability, morbidity, and mortality [16,17,18,19,20,21]. Recently, reputable magazine reports have mentioned a potential association between obesity and hookah smoking; however, this is still uncertain [22]. Moreover, to the best of our knowledge, no systematic review considering this issue as a primary outcome has yet been conducted in order to provide a valid interpretation of the evidence published to date based on a systematic review and a meta-analysis. In light of these considerations, we hypothesised an association between hookah smoking and a higher risk of obesity and aimed to systematically review the published literature on this topic in accordance with the PICO process [23], as detailed below:

P—Population: adolescents and adults of both genders [24]; I—Intervention: active hookah smoking; C—Comparison: hookah-smoking group vs. nonsmoking group (when available) or hookah-smoking group vs. cigarette-smoking group (when available); and O—Outcome: obesity, however defined, based on international guidelines, (e.g., BMI, BMI percentiles, waist circumference, body fat percentage, etc.).

## 2. Methods

The current study was completed according to the Preferred Reporting Items for the Systematic reviews and Meta-Analyses (PRISMA) guidelines [25,26] and registered in the PROSPERO registry, York, UK—Association between smoking shisha, obesity, and related comorbidities: a systematic review (CRD42019129389) [27].

### 2.1. Inclusion and Exclusion Criteria

All studies evaluating hookah smoking and obesity were included, provided they met the following criteria: (i) they were written in English, (ii) they were original articles, and (iii) they related to prospective or retrospective observational (analytical or descriptive), experimental, or quasi-experimental controlled or noncontrolled studies. Reviews or non-original articles (e.g., case reports, editorials, letters to editors, or book chapters) were excluded.

### 2.2. Information Source and Search Strategy

The literature search was designed and performed independently in duplicate by two of the authors: the principal and the senior investigator. The PubMed/MEDLINE database was systematically screened using the following MeSH terms: #1 = Obesity, #2 = Hookah, #3 = Water pipe, #4 = Narghile, #5 = Arghile, and #6 = Shisha, together with the combinations #1 AND #2 OR #3 OR #4 OR #5 OR #6. In addition, a manual search was carried out to retrieve other articles that had not been identified via the initial search strategy. The publication date was not considered as an exclusion criterion for the purposes of this review.

### 2.3. Study Selection

Two authors independently screened the resulting articles for their methodologies and appropriateness for inclusion. All the included studies underwent a risk-of-bias assessment according to the 10-item quality assessment checklist for prevalence studies adapted by Hoy and colleagues, in which a total score of 0–3 indicates a low risk of bias, a score of 4–6 indicates a moderate risk of bias, and a score of 7–9 indicates a high risk of bias [28]. Consensus discussions were used to resolve disagreements between reviewers.

### 2.4. Data Collection Process and Data Items

The title and abstract of each paper were firstly assessed by two independent authors for language suitability and subject-matter relevance, and the studies selected were assessed in terms of their appropriateness for inclusion and the quality of the method. Those studies passing both rounds of screening are shown in Table 1.

### 2.5. Data Synthesis

The studies that met the inclusion criteria have been presented as a narrative synthesis [29,30]. Subsequently, a meta-analysis was conducted, detecting the association between hookah smoking and the risk of obesity, however expressed, using Review Manager 5 (RevMan 5.3. Copenhagen, Denmark) software developed by and for the Cochrane collaboration [31]. A random effects model was used to calculate the pooled relative risk and the 95% CI.

## 3. Results

**Figure 1:**
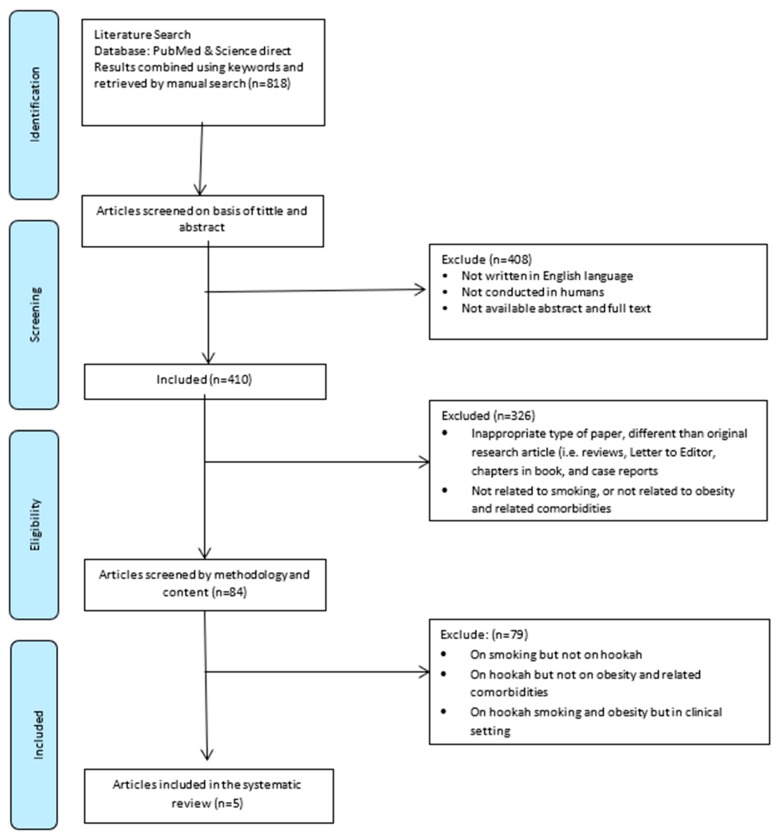
The flowchart summarizing the study selection procedure.

The initial search retrieved 818 papers. After the first round of screening (titles and abstracts), 408 papers were excluded on the following grounds: They were not in English or did not study humans, or the abstracts and full texts were not available. The second round of screening excluded articles (n = 326) that represented an inappropriate type of paper, were not an original research article, (e.g., reviews, letters to editors, book chapters, and case reports), or were not related to smoking or obesity and related comorbidities. Of the remaining 84 articles dealing with smoking and health status, a further 79 papers were excluded on the following grounds: They were on smoking but not on hookahs, they considered health outcomes other than obesity and related comorbidities (e.g., cancer, respiratory diseases, acute effects of hookah-smoking such as heart rate, etc.), or other factors, (e.g., they were conducted in clinical settings rather than in the general population). Thus, at the end of the screening process, five articles were available for systematic review, narrative synthesis, and meta-analysis (Figure 1). According to the quality assessment checklist for prevalence studies (n = 5), these studies had a low risk of bias (mean score of 1.2 points) (Table 2).

### 3.1. Narrative Synthesis

In 2012, Shafique et al. [32] conducted a cross-sectional population-based study to investigate the association between hookah smoking and metabolic syndrome as a primary outcome. The sample included 2032 individuals, of which 325 were current hookah smokers. Metabolic syndrome was significantly higher among the current hookah smokers (33.1%) compared to nonsmokers (14.8%); the former were three times more likely to have metabolic syndrome compared with nonsmokers after an adjustment for confounders. Moreover, the definition of obesity was based on waist circumference. For abdominal obesity, the authors used a South Asian-specific cutoff of ≥90 cm waist circumference for males and of ≥80 cm for females [33]. In fact, hookah smokers had a significantly greater waist circumference (84.7 ± 12.6 vs. 80.6 ± 11.8; *p* < 0.01), and a logistic regression analysis showed that hookah smokers were significantly more likely to show abdominal obesity (OR 1.93, 95% CI 1.52–2.45).

In 2015, Ward et al. [34] conducted a population-based household study among 2536 adults (age ≥ 18 years) and examined the associations between hookah smoking and BMI and obesity status (BMI ≥ 30 kg/m^2^). Of the total sample 2134 had never smoked a hookah, 116 were former smokers, 251 were current non-daily smokers, and 35 were current daily smokers. The mean BMI of the entire sample was 30.2 ± 6.3 kg/m^2^. The authors found that daily hookah smokers had a BMI nearly 2 units greater than nonsmokers and had nearly three times the risk of obesity. 

In 2018, Saffar Soflaei et al. [35] published a large population study with a total of 9840 subjects living in the city of Mashad (Iran), allocated to five different groups: nonsmokers (n = 6742), ex-smokers (n = 976), cigarette smokers (n = 864), hookah smokers (n = 1067), and cigarette and hookah smokers (n = 41). The authors found a significant association between hookah smoking (not cigarette-smoking) and obesity. They concluded that, in contrast to the common belief that the hookah eliminates the toxicity of tobacco compared with cigarettes, the adverse effects of hookah smoking could be even greater than those of cigarette smoking. In fact, in this study, the prevalence of obesity was significantly higher in hookah smokers compared with nonsmokers and even cigarette smokers.

In 2018, Alomari et al. [36] studied the associations between obesity and hookah smoking among 2313 adolescents of both genders at public schools in grades seven to 10 in Jordan using a cross-sectional design. The BMI percentile z-scores were calculated to determine weight-status categories, and obesity was defined as the 95th percentile or greater. Of the entire sample, 279 (12.1%) were obese. The authors found that body weight and age- and gender-specific BMI were higher for hookah smokers compared to nonsmokers and that those who smoked a hookah weekly had double the odds of being obese compared to nonsmokers (OR = 2.14; 95% CI = 1.08–4.21; *p* = 0.028). They concluded that hookah use and dual use are associated with greater obesity, BMI, and body weight among Jordanian adolescents.

In 2018, Hasni et al. [37] undertook a small population study that aimed to compare the biochemical and metabolic profiles of hookah smokers and nonsmokers in 58 young males aged between 25 and 45 with no known history of metabolic or cardiovascular diseases. Abdominal obesity was defined based on the International Diabetes Federation (IDF) criteria, i.e., WC ≥ 94 cm [38], and obesity was defined as BMI ≥ 30 kg/m^2^. The mean BMI in hookah smokers was significantly higher than that of nonsmokers (28.2 ± 3.6 vs. 26.5 ± 2.6; *p* = 0.046), and there was a higher prevalence of obesity (37.9% vs. 6.9%; *p* = 0.04) and a higher prevalence of abdominal obesity (79.3% vs. 59.6%; *p* = 0.08) among hookah smokers.

### 3.2. Meta-Analysis

**Figure 2:**
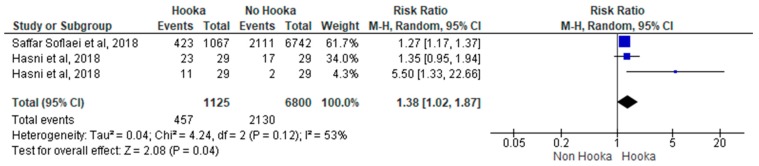
The forest plot for the risk of obesity with hookah-smoking.

The meta-analysis results estimating the overall risk ratios for obesity in hookah smokers compared to nonsmokers are presented in Figure 2. The random effect weighted pooled risk for obesity in hookah smokers indicated an increased risk of obesity of approximately 38%, compared to nonsmokers (RR = 1.38; 95% CI = 1.02–1.87; *p* = 0.04). The heterogeneity analysis revealed a moderate variability (I^2^ = 53%).

## 4. Discussion

The aim of the current systematic review was to provide benchmark data on the association between hookah smoking and obesity. Five studies, comprising a total of 16,779 adolescent and adult participants and age range between 13–75 years and conducted in Iran, Syria, Jordan, Pakistan, and Tunisia, met the inclusion criteria and were reviewed, revealing one major finding: All five studies included in our systematic review showed a higher prevalence of obesity and/or a higher association between obesity (abdominal obesity, BMI percentile ≥ 95th, or BMI ≥ 30 kg/m^2^) and hookah smoking than the corresponding values for nonsmokers and cigarette smokers (when comparisons were available) regardless of gender and among all ages. This finding is considered to be strong and robust because (i) data were derived from well-conducted, large-sample, population-based studies with a low risk of bias; (ii) the finding was not contradicted in any of the included studies; (iii) the same finding has also been reported in clinical samples (i.e., not the general population) [39]; and (iv) this finding was confirmed by a meta-analysis. 

### 4.1. Clinical Implications

Our findings have some implications, especially for the general population. Firstly, it is important to discuss the association between hookah smoking and obesity among young adults, perhaps through educational interventions in schools and universities and in work settings [40,41]. In addition, the common public belief that hookah smoking may be healthy, since hookah smoke contains fruit flavours and the water in the bottom of the hookah can eliminate the toxicity of tobacco compared with cigarettes, should be contradicted. On the contrary, we found that the adverse effects of hookah smoking could be even greater than those of cigarette smoking. In fact, several types of cancer (e.g., lung cancer) have been linked to hookah smoking [42]. Moreover, it causes coronary artery disease [39], an increased heart rate and high blood pressure [43], respiratory diseases [10], dental problems [44], and osteoporosis [45], as well as infections when sharing a hookah [45].

It is unclear why smoking hookah is associated with obesity; we speculate that the potential mechanisms behind this association may be multiple. However, two factors may have a major impact. Firstly, smoking a hookah requires sitting, and a hookah-smoking session may last for two hours. Some individuals may repeat the session two or three times a day [46], and this unavoidably facilitates a sedentary lifestyle (unlike cigarettes), which reduces energy expenditure [47]. Also, the hookah is smoked during social events where smokers spend time together and talk as they pass the mouthpiece around in environments (e.g., restaurants and coffee shops) rich in eating stimuli, which could increase the exposure to and consumption of high-calorie foods [47]. All in all, it has been shown that hookah smoking is associated with less healthy lifestyle habits in both men and women [48].

### 4.2. Strengths and Limitations

This systematic review has certain strengths. To the best of our knowledge, this is the first systematic review to investigate the association between hookah smoking and obesity. Despite the fact that few studies met the inclusion criteria and were included in our systematic review, the finding is considered to be strong, with definite evidence for the association between hookah smoking and obesity. This needs to be underlined due to the increasing trend of this smoking habit, especially among young people. However, this systematic review also has certain limitations. In particular, our results should be interpreted with caution with regard to the association between hookah smoking and obesity, since the cross-sectional design of the studies included in our systematic review indicates only simple associations at best and does not provide solid information regarding any causal relationships between conditions [49]. In other words, these studies lack evidence to determine whether hookah smoking may lead to obesity, since very few studies have longitudinally investigated the “real” effects of hookah smoking [50]. Moreover, the included studies in our systematic review were conducted only in low-middle income countries (i.e., Middle East); therefore, our findings may not be generalized on a global scale. Finally, none of the included studies clearly examined if the average number of sessions (i.e., per day or week) or years (i.e., months and years) of hookah smoking are related to a higher risk of obesity. All these shortcomings in the current research indicate the need to design longitudinal studies to clarify the real effect of hookah smoking on the onset and progression of obesity and weight-related comorbidities, especially in Western countries (i.e., US and Europe).

## 5. Conclusions

Despite the scarcity of studies, the preliminary findings indicate a high prevalence of obesity in hookah smokers. Public health policymakers should be aware of this for the better management of obesity and other diseases related to hookah smoking. 

## Figures and Tables

**Table 1:** Summary of the included studies.

Study	Design	Country	Sample	Age	Primary Outcome	Findings
Shafique et al. 2012	Population-based study	Pakistan	Total = 2032 HS = 325 of both genders	30–75 years	• Association between HS and metabolic syndrome and components	• Metabolic syndrome was significantly higher among HS (33.1%) compared to NS. • HS were 3 times more likely to have metabolic syndrome compared with NS. • HS have significantly more hypertriglyceridemia, hyperglycaemia, hypertension, and abdominal obesity with respect to non-HS.
Ward et al. 2015	Population-based study	Syria	Total = 2536, NS = 2134, former HS = 116, 251 non-daily HS = 251, daily HS = 35 of both genders	≥18 years	• Associations of HS use status with BMI and obesity status	• Daily HS have nearly 2 BMI units greater than NS and had nearly three times the risk of having obesity.
Saffar Soflaei et al. 2018	Population-based study	Iran	Total = 9840, NS = 6742, Ex-smoker = 976 CS = 864, HS = 1067, MS = 41 of both genders	35–65 years	Association between HS and obesity, cardiovascular disease, diabetes mellitus, metabolic syndrome, and dyslipidemia	• A positive association between HS and metabolic syndrome, diabetes, obesity, and dyslipidemia was not established in CS.
Alomari et al. 2018	Population-based study	Jordan	Total = 2313 of both genders	In grades 7–10	• Associations of obesity with HS	• HS when compared to nonusers and who smoked hookah weekly had twofold greater odds of having obesity than nonsmokers.
Hasni et al. 2018	Population-based study	Tunisia	Total = 58, HS = 29, NS = 29 only males	25–45 years	• Comparison in the biochemical data and the metabolic profile between HS and nonsmokers	• The mean BMI in HS was significantly higher when compared with that of nonsmokers and had a higher prevalence of obesity and abdominal obesity.

HS = hookah smokers; NS = nonsmokers; CS = cigarette-smokers; BMI = body mass index.

**Table 2:** Quality assessment checklist for prevalence studies.

	Shafique 2012	Ward 2015	Saffar Soflaei 2018	Alomari 2018	Hasni 2018
Was the study’s target population a close representation of the national population in relation to relevant variables, e.g., age, sex, occupation?	0	0	0	0	1
Was the sampling frame a true or close representation of the target population?	0	0	0	0	1
Was some form of random selection used to select the sample, OR was a census undertaken?	0	0	0	1	1
Was the likelihood of nonresponse bias minimal?	0	1	0	0	1
Were data collected directly from the subjects as opposed to a proxy?	0	0	0	0	0
Was an acceptable case definition used in the study?	0	0	0	0	0
Was the study instrument that measured the parameter of interest shown to have reliability and validity (if necessary)?	0	0	0	0	0
Was the same mode of data collection used for all subjects?	0	0	0	0	0
Were the numerator(s) and denominator(s) for the parameter of interest appropriate?	0	0	0	0	0
Summary on the overall risk of study	0	1	0	1	4

Yes = 0; No = 1; Total score 0–3 = low risk of bias; 4–6 = moderate risk of bias; 7–9 = high risk of bias
